# Prevalence and determinants of parental refusal of human papillomavirus vaccination in Morocco: A multicenter cross-sectional study

**DOI:** 10.1371/journal.pone.0348586

**Published:** 2026-05-06

**Authors:** Touria Essayagh, Meriem Essayagh, Kaoutar Nmila, Housnia Slibani, Soukayna El Hachimi, Mustapha El Boukhari, Hajar Lemriss, Sanaâ Lemriss, Rehmatullah Zadran, Halima Tigaidi, Sanah Essayagh

**Affiliations:** 1 Hassan First University of Settat, Institut Supérieur des Sciences de la Santé, Laboratoire des Sciences et ingénierie biomédicales, biophysique et santé, Settat, Morocco; 2 Office National de Sécurité Sanitaire des produits Alimentaires, Meknès, Morocco; 3 Laboratoire of Research and Medical Analysis of Gendarmerie Royale, Department of Biosafety, Rabat, Morocco; 4 National Infectious Diseases Hospital Laboratory, Kabul, Afghanistan; 5 University of Kordofan, Faculty of Public and environmental Health, Elobeid, Sudan; 6 Hassan First University of Settat, Faculté des Sciences et Techniques, Laboratoire Agroalimentaire et Santé, Settat, Morocco; Federal University Otuoke, NIGERIA

## Abstract

**Introduction:**

Vaccination against the human papillomavirus (HPV) is an effective way to avert cervical cancer. However, acceptance in Morocco remains inadequate. The aim of this study was to measure the prevalence of parental HPV vaccine refusal and the risk factors associated with it.

**Methods:**

Between 3 March and 30 September 2025, a multicenter cross-sectional survey was conducted among Moroccan parents of girls aged 11–14 who attended health facilities. The study looked at sociodemographic factors, knowledge regarding HPV and cervical cancer, and vaccination attitudes. A multivariate logistic regression analysis was used to identify risk factors for vaccination refusal.

**Results:**

The research included 1,444 participants with an average age of 37.7 ± 6.6 years. Of them, 415 refused HPV vaccination for their daughters, resulting in a prevalence of 28.7%. Vaccine refusal was substantially related to higher educational levels, lower income, less faith in the healthcare system, less knowledge about cervical cancer and its symptoms, and insufficient information regarding the HPV vaccine as seen by the media. Refusal was also associated with a poor perception of the seriousness of HPV infection, fear of vaccinating daughters, noncompliance with past vaccination schedules, difficulties accessing health centers, and a lack of recommendations from healthcare professionals. In contrast, refusal was inversely associated with parents who were uninformed of the proper number of vaccination doses or uninformed regarding the availability of the HPV vaccine at health centers or who feared the vaccine may cause an adverse reaction.

**Conclusion:**

Parental refusal of HPV vaccination is still a substantial obstacle. To increase acceptability and enhance cervical cancer prevention in Morocco, it is critical to expand communication strategy, boost public knowledge, and assure active participation of healthcare professionals.

## Introduction

Human papillomavirus (HPV) infection remains the principal etiological factor in the development of cervical cancer, a disease largely attributable to persistent infection with high-risk genotypes, particularly HPV types 16 and 18. When these infections are not cleared, they may progress from precancerous lesions to invasive cancer over time [1]. In 2022, globally, this cancer ranked fourth among the most common cancers in women, with a total of 660,000 new cases reported and 350,000 deaths. Ninety-four percent of these deaths occurred in low- and middle-income countries [2,3]. HPV vaccination is the primary method for reducing the morbidity and mortality associated with this disease [4].

In the countries of the Eastern Mediterranean Region, age-standardized incidence rates are less than 10 per 100,000 women, with the exception of Somalia, where the age-standardized incidence rate is 25.4 per 100,000; Djibouti, where it is 13.4 per 100,000; and Afghanistan and Morocco, where it is 10.4 per 100,000 for each country [5,6].

In line with the World Health Organization’s framework for eliminating cervical cancer as a public health problem and Morocco’s national cancer prevention and control plan, it has been agreed to ensure that at least 90% of girls aged 11 years are vaccinated against HPV by 2030 [7,8].

In October 2022, a quadrivalent HPV vaccine was introduced into Morocco’s national immunization program. Vaccination is targeted at 11-year-old girls attending school and is administered in two doses at least six months apart (Gardasil) [9]. This strategy relied on the healthcare system’s capacity to integrate a new vaccine and drew on the successful experiences of several countries that had identified factors contributing to high-performing HPV vaccination programs.

However, the effectiveness of this strategy is highly dependent on parental acceptance of the vaccine. Yet, HPV vaccination—although effective, safe, and available—faces significant obstacles in many contexts, including parental refusal. The determinants of this refusal are numerous, including a lack of knowledge about the disease, a low perception of risk, fears related to the vaccine, institutional distrust, socioeconomic factors, and the absence of explicit recommendations from healthcare professionals [10–12].

In this context, it was important to identify, among the Moroccan population, the factors associated with parental refusal of HPV vaccination for their daughters in order to develop appropriate strategies. The objective of our study is therefore to estimate the prevalence of parental refusal of HPV vaccination and to identify the associated risk factors. The results of this study are essential to better guide public health policies and health strategies in order to improve HPV vaccination coverage and, ultimately, reduce the incidence of cervical cancer in women in Morocco and improve their sexual and reproductive health.

## Methods

### Setting study

Morocco, a country of 37 million people, including around 3.5 million children aged 10–14, is located in the northwest of Africa, bordering the Mediterranean Sea to the north, the Atlantic Ocean to the west, Algeria to the east, and Mauritania to the south. The healthcare system is divided into 12 regions, 82 provinces, and 2,205 health centers [13].

### Study design and population

Between March 3 and September 30, 2025, we conducted a multicenter cross-sectional survey of parents of girls aged 11–14 years attending health centers in the provinces of Guercif, Tinghir, Kenitra, Khouribga, and Settat. A multi-stage cluster random sampling method was used. First, five health regions were randomly selected from among the twelve regions of Morocco (Oriental, Drâa-Tafilalet, Rabat-Salé-Kenitra, Béni Mellal-Khenifra, and Casablanca-Settat). Second, one province was randomly selected within each region. Third, health centers were randomly selected within each province. At each selected center, mothers, fathers, or legal guardians presenting for consultations or routine vaccinations on the survey days were invited to participate after providing written informed consent. Only one parent per child was interviewed to avoid redundant responses. Eligibility was verified by asking participants if they had a daughter between the ages of 11 and 14. Parents whose daughters had previously been sexually active and those whose daughters had a medical contraindication to HPV vaccination were excluded. Sexual activity status was based solely on parental self-reporting.

### Sample size determination

Given an estimated prevalence of parental refusal of HPV vaccination of 22.1% and a margin of error of 5%, the minimum sample size was set at 265 participants per province. This calculation was performed using Epi-Info 7 software, in accordance with epidemiological methodological standards for estimating a proportion in the population.

### Data collection

Data were collected using a structured questionnaire administered during face-to-face interviews between the interviewer and the parents of girls aged 11–14 years. This questionnaire included sociodemographic information and assessed knowledge levels related to HPV, cervical cancer, and the vaccine. It also investigated parental perceptions of disease severity, vulnerability to HPV infection, and perceived benefits, potential risks, and barriers to HPV vaccination. All items were developed based on the Health Belief Model [14].

### Operational definitions

Refusal of HPV vaccination was assessed based on parental decisions regarding their daughter’s vaccination. Parents were first asked about their daughter’s HPV vaccination status. If the answer was negative, a second question concerned their intention to vaccinate her. Parents who stated they did not intend to vaccinate their daughter were classified as “refusing vaccination,” while those planning future vaccination or citing temporary constraints were classified as “not refusing.”

Perceived susceptibility to HPV infection was assessed based on participants’ self-reported risk of infection. Perceived severity was assessed according to participants’ stated beliefs about the seriousness of the health threat posed by HPV.

The perceived benefits of vaccination were assessed by asking participants about the vaccine’s supposed effectiveness in preventing infection. Perceived risks associated with vaccination were estimated through statements regarding the vaccine’s safety and the absence of expected adverse effects. Perceived barriers were measured by the degree of concern expressed about the vaccine’s potential impact on certain adolescent behaviors. The influence of social norms was assessed by participants’ self-reported influence on their social environment regarding vaccination decisions. Fear of regret was explored using two binary (yes/no) items addressing (i) fear of getting vaccinated and (ii) fear of regretting vaccination. Prior vaccination behavior was measured using two binary (yes/no) items addressing (i) prior receipt of vaccines from the national vaccination schedule and (ii) prior refusal of a recommended vaccine. Finally, access to healthcare was assessed by asking participants about the distance, travel time, and means of transportation required to reach the nearest health center [14–17].

### Data management and statistical analysis

We used Epi Info software, version 7.2.0.1, to analyze the data. A statistical significance level of 0.05 was set for all two-sided tests. We described quantitative variables as means and standard deviations, while qualitative variables were expressed as numbers and percentages. A bivariate analysis was conducted to identify factors associated with vaccination refusal. Variables with a p-value ≤ 0.05 at this stage were included in a multivariate analysis. A preliminary descriptive analysis was performed on the entire study population, followed by subgroup analyses.

The relevance of associations involving qualitative variables was assessed using Pearson’s chi-squared test, while Student’s t-test was used for comparisons involving quantitative variables. Finally, the association between each potential risk factor and refusal of HPV vaccination was estimated using the odds ratio (OR) and its 95% confidence interval.

### Ethical considerations

Before completing the questionnaires, all participants were informed of the study’s objectives and data collection methods. In accordance with the ethical principles set forth in the Declaration of Helsinki, participants were asked to sign an informed written statement of consent. To protect the rights and dignity of each participant, data confidentiality and anonymity were rigorously guaranteed. To ensure the study’s compliance with national and international ethical standards, the research protocol (reference no. 35/24) was reviewed and approved by the Ethics Committee of Mohammed V University in Rabat, Morocco. The committee approved the use of parental self-report for sensitive items including sexual activity status.

## Results

### 1. Socioeconomic and demographic characteristics

A total of 1,444 participants were included in the study, and 415 (28.7%) refused to have their daughters vaccinated against HPV. The mean age of the participants was 37.7 ± 6.6 years, and 1,321 (91.5%) were married. Furthermore, 754 (52.2%) participants had a monthly household income of less than $299, 268 (18.6%) spent more than 30 minutes traveling to the health center, and 710 (49.2%) reported using public transportation to get there. Finally, 668 participants (46.2%) reported having little or no confidence in the healthcare system “Table 1”.

**Table 1:** The socioeconomic and demographic characteristics of the parents, Morocco, 2025.

Quantitative variables		Total	Refusal of the HPV vaccine	Acceptability of the HPV vaccine	*P-value*
Participants’ total no. (%)		1,444 (100)	415 (28.7)	1,029 (71.3)	
Age in years±sd		37.7 ± 6.6	38.2 ± 6.7	37.6 ± 6.5	0.41
**Qualitative variables no. (%)**					
Sex					0.28
	Male	92 (06.4)	31 (07.5)	61 (05.9)	
	Female	1,352 (93.6)	384 (92.5)	968 (94.1)	
Area of residence					<0.001
Rural		683 (47.3)	226 (54.5)	457 (44.4)	
Urban		761 (52.7)	189 (45.5)	572 (55.6)	
Marital status					0.048
	Uncoupled^*†*^	123 (08.5)	45 (10.8)	78 (07.6)	
	Coupled^*^	1,321 (91.5)	370 (89.2)	951 (92.4)	
Level of education					0.001
	University	556 (38.5)	134 (32.3)	422 (41.0)	
	High school	310 (21.5)	81 (19.5)	229 (22.2)	
	Middle school	290 (20.1)	97 (23.4)	193 (18.8)	
	Elementary	215 (14.8)	79 (19.0)	136 (13.2)	
	Illiteracy	73 (05.1)	24 (05.8)	49 (04.8)	
Level of education conjoint					0.0003
	University	495 (34.2)	128 (30.8)	367 (35.7)	
	High school	266 (18.4)	55 (13.3)	211 (20.5)	
	Middle school	226 (15.7)	76 (18.3)	150 (14.6)	
	Elementary	248 (17.2)	91 (21.9)	157 (15.3)	
	Illiteracy	209 (14.5)	65 (15.7)	144 (13.9)	
Monthly income per household ($)					<0.001
	<199	328 (22.7)	142 (34.2)	186 (18.0)	
	200-299	426 (29.5)	110 (26.5)	316 (30.7)	
	300-499	384 (26.6)	86 (20.7)	298 (29.0)	
	≥500	306 (21.2)	77 (18.6)	119 (22.3)	
Time to arrive at the health center					0.0004
	More than one hour	33 (02.3)	19 (04.6)	14 (01.4)	
	Between half an hour and an hour	235 (16.3)	77 (18.6)	158 (15.4)	
	Less than half an hour	1,176 (81.4)	319 (76.8)	857 (83.2)	
Distance from the health center (kilometer)					0.16
	More than ten	90 (06.2)	31 (07.5)	59 (05.7)	
	Between six and ten	278 (19.3)	69 (16.6)	209 (20.3)	
	Less than six	1,076 (74.5)	315 (75.9)	761 (74.0)	
Type of transport					0.006
	By transport	710 (49.2)	228 (54.9)	482 (46.8)	
	On foot	734 (50.8)	187 (45.1)	547 (53.2)	
Trust in the healthcare system					<0.001
	No	194 (13.4)	147 (35.4)	47 (04.6)	
	A little	474 (32.8)	191 (46.0)	283 (27.5)	
	Yes	776 (53.8)	77 (18.6)	699 (67.9)	

sd stands for standard deviation. *: Coupled refers to being married. †: Uncoupled means single, divorced, or widowed. HPV stands for human papillomavirus. When the requirements were met, the Pearson chi-squared test determined the relationship between the dependent and independent variables. For the quantitative variables, we employed an independent samples t-test; a p-value of less than 0.05 was considered significant.

### 2. Knowledge about cervical cancer and the human papillomavirus vaccine

Table 2 presents parents’ knowledge regarding cervical cancer and the HPV vaccine. Overall, 989 participants (68.5%) had never heard of HPV, while 487 (33.7%) reported no prior awareness of cervical cancer. Unsatisfactory general knowledge about cervical cancer was observed among 886 participants (61.4%). In addition, 828 participants (57.3%) demonstrated inadequate knowledge of risk factors, 1,021 (70.7%) of symptoms, 1,232 (85.3%) of complications, and 854 (59.1%) of preventive measures. Regarding the HPV vaccine, 1,201 participants (83.2%) were unaware of the recommended number of doses. Furthermore, 1,112 participants (77.0%) reported an insufficient perception of the level of information on the HPV vaccine disseminated by the media “Table 2”.

**Table 2:** General knowledge about cervical cancer and the human papillomavirus vaccine, parents, Morocco, 2025.

Qualitative variables		Total (n = 1,444)	Refusal of the HPV vaccine (n = 415)	Acceptability of the HPV vaccine (n = 1,029)	*P-value*
Having already heard of HPV infection					<0.001
	No	989 (68.5)	317 (76.4)	672 (65.3)	
	Yes	455 (31.5)	98 (23.6)	357 (34.7)	
Having already heard of cervical cancer					<0.001
	No	487 (33.7)	208 (50.1)	279 (27.1)	
	Yes	957 (66.3)	207 (49.9)	750 (72.9)	
General knowledge about cervical cancer					<0.001
	Unsatisfactory	886 (61.4)	333 (80.2)	553 (53.7)	
	Satisfactory	558 (38.6)	82 (19.8)	476 (46.3)	
Knowledge of the risk factors for cervical cancer					<0.001
	Unsatisfactory	828 (57.3)	305 (73.5)	523 (50.8)	
	Satisfactory	616 (42.7)	110 (26.5)	506 (49.2)	
Knowledge of the symptoms of cervical cancer					<0.001
	Unsatisfactory	1,021 (70.7)	371 (89.4)	650 (63.2)	
	Satisfactory	423 (29.3)	44 (10.6)	379 (36.8)	
Knowledge of the complications of cervical cancer					<0.001
	Unsatisfactory	1,232 (85.3)	384 (92.5)	848 (82.4)	
	Satisfactory	212 (14.7)	31 (07.5)	181 (17.6)	
Knowledge of preventive measures against cervical cancer					<0.001
	Unsatisfactory	854 (59.1)	317 (76.4)	537 (52.2)	
	Satisfactory	590 (40.9)	98 (23.6)	492 (47.8)	
Knowledge of the target population					0.08
	Unsatisfactory	952 (65.9)	288 (69.4)	664 (64.5)	
	Satisfactory	492 (34.1)	127 (30.6)	365 (35.5)	
Knowledge about the number of doses of the HPV vaccine					0.001
	Unsatisfactory	1,201 (83.2)	324 (78.1)	877 (85.2)	
	Satisfactory	243 (16.8)	91 (21.9)	152 (14.8)	
Perception of the level of information provided by the media on the HPV vaccine					<0.001
	Unsatisfactory	1,112 (77.0)	348 (83.9)	764 (74.2)	
	Satisfactory	332 (23.0)	67 (16.1)	265 (25.8)	

*HPV stands for human papillomavirus. When the requirements were met, the Pearson chi-squared test determined the relationship between the dependent and independent variables. For the quantitative variables, we employed an independent samples t-test; a p-value of less than 0.05 was considered significant.*

### 3. Beliefs and fears about the human papillomavirus vaccine

Among the 1,444 participants, 939 (65.0%) believed that their daughters were unlikely to contract HPV. A total of 303 participants (21.0%) underestimated the severity of HPV infection, while 396 (27.4%) recognized the benefits of HPV vaccination. However, 846 participants (58.6%) feared potential side effects of the vaccine, and 461 (31.9%) worried that vaccination might encourage early sexual activity. Nearly half of the parents, 693 (48.0%), felt afraid to vaccinate their daughters, and 661 (45.8%) were concerned that they might later regret the decision “Table 3”.

**Table 3:** Parents’ beliefs and fears regarding the human papillomavirus vaccine, parents, Morocco, 2025.

Qualitative variables		Total (n = 1,444)	Refusal of the HPV vaccine (n = 415)	Acceptability of the HPV vaccine (n = 1,029)	*P-value*
Susceptibility to HPV infection					<0.001
	No	939 (65.0)	198 (47.7)	741 (72.0)	
	Yes	505 (35.0)	217 (52.3)	288 (28.0)	
Perceived beliefs about the seriousness of HPV infection					<0.001
	No	303 (21.0)	168 (40.5)	135 (13.1)	
	Yes	1,141 (79.0)	247 (59.5)	894 (86.9)	
Perceived beliefs about HPV vaccine benefits					<0.001
	No	396 (27.4)	205 (49.4)	191 (18.6)	
	Yes	1,048 (72.6)	210 (50.6)	838 (81.4)	
HPV vaccine adverse reaction					<0.001
	Yes	846 (58.6)	107 (25.8)	739 (71.8)	
	No	598 (41.4)	308 (74.2)	290 (28.2)	
HPV vaccine would encourage sexual activity					0.015
	Yes	461 (31.9)	113 (27.2)	348 (33.8)	
	No	983 (68.1)	302 (72.8)	681 (66.2)	
**Fear of regret over HPV vaccination**					
Parents’ fear of having their young girls vaccinated with the anti-HPV vaccine					<0.001
	Yes	693 (48.0)	310 (74.7)	383 (37.2)	
	No	751 (52.0)	105 (25.3)	646 (62.8)	
Parents’ fear of regretting having vaccinated their young girls with the anti-HPV vaccine					<0.001
	Yes	661 (45.8)	249 (60.0)	412 (40.0)	
	No	783 (54.2)	166 (40.0)	617 (60.0)	

*HPV stands for human papillomavirus. When the requirements were met, the Pearson chi-squared test determined the relationship between the dependent and independent variables. For the quantitative variables, we employed an independent samples t-test; a p-value of less than 0.05 was considered significant.*

### 4. Environnemental influences

Table 4 presents the influence of environmental factors on the decision to vaccinate against HPV. 960 parents (66.5%) felt that the media had a negative influence on their vaccination decision, while 872 (60.4%) reported that societal norms also had a negative impact on their choice. Furthermore, 1007 participants (69.7%) reported having received no recommendation from a healthcare professional regarding HPV vaccination for their daughters “Table 4”.

**Table 4:** Influence of environmental factors on the decision to vaccinate against human papillomavirus, parents, Morocco, 2025.

Qualitative variables		Total (n = 1,444)	Refusal of the HPV vaccine (n = 415)	Acceptability of the HPV vaccine (n = 1,029)	*P-value*
Media influence on HPV vaccination					0.001
	Negative	960 (66.5)	302 (72.8)	658 (64.0)	
	Positive	484 (33.5)	113 (27.2)	371 (36.0)	
Influence of societal norms on HPV vaccination					<0.0001
	Negative	872 (60.4)	307 (74.0)	565 (54.9)	
	Positive	572 (39.6)	108 (26.0)	464 (45.1)	
Influence of previous experiences with cancer					
Have already been in contact with a person suffering from cancer					0.02
	No	1,177 (81.5)	354 (85.3)	823 (80.0)	
	Yes	267 (18.5)	61 (14.7)	206 (20.0)	
Have already had sexual diseases					0.86
	No	1,401 (97.0)	402 (96.9)	999 (97.1)	
	Yes	43 (03.0)	13 (03.1)	30 (02.9)	
Previous vaccination behavior					
Have already vaccinated your children previously according to the vaccination schedule					<0.001
	No	108 (07.5)	79 (19.0)	29 (02.8)	
	Yes	1,336 (92.5)	336 (81.0)	1,000 (97.2)	
Have refused to comply with all vaccinations recommended for their children in the past					0.02
	Yes	7 (00.5)	5 (01.2)	2 (00.2)	
	No	1,437 (99.5)	410 (98.8)	1,027 (99.8)	
The doctor or nurse informs the parents that the health center has a vaccine against HPV					<0.001
	No	1,029 (71.3)	343 (82.6)	686 (66.7)	
	Yes	415 (28.7)	72 (17.4)	343 (33.3)	
The doctor or nurse informs the parents about the free HPV vaccine at the health center					<0.001
	No	1,112 (77.0)	348 (83.9)	764 (74.3)	
	Yes	332 (23.0)	67 (16.1)	265 (25.7)	
The doctor or nurse advises parents to vaccinate their daughters against HPV at the health center					<0.001
	No	1,007 (69.7)	351 (84.6)	656 (63.8)	
	Yes	437 (30.3)	64 (15.4)	373 (36.2)	

*HPV stands for human papillomavirus. When the requirements were met, the Pearson chi-squared test determined the relationship between the dependent and independent variables. For the quantitative variables, we employed an independent samples t-test; a p-value of less than 0.05 was considered significant.*

### 5. Bivariate analysis

Among the parents, 415 participants (28.7%) reported refusing the HPV vaccine for their daughters. In the bivariate analysis, a significance threshold of p ≤ 0.05 was applied, identifying 32 factors significantly associated with vaccine refusal. Sociodemographic factors included area of residence (p = 0.0005), being uncoupled (p = 0.045), the participant’s level of education (p = 0.001), the spouse’s level of education (p = 0.0003), and low monthly household income (p < 0.001). Access to healthcare, such as travel time to the health center (p = 0.0005) and reliance on transport (p = 0.005), was also associated with refusal. Participants with low trust in the healthcare system (p < 0.001), those who had never heard of HPV (p < 0.001) or cervical cancer (p < 0.001), and those who had prior contact with a person with cervical cancer (p = 0.01) were more likely to refuse vaccination. Insufficient knowledge about cervical cancer, including risk factors (p < 0.001), symptoms (p < 0.001), complications (p < 0.001), preventive measures (p < 0.001), and the number of HPV vaccine doses (p = 0.0013), was also significantly associated with refusal. Furthermore, participants who perceived media coverage about the HPV vaccine as unsatisfactory (p = 0.0001), who underestimated their daughters’ susceptibility to HPV (p < 0.001), who believed the infection was not severe (p < 0.001), or who doubted the vaccine’s effectiveness (p < 0.001) were more likely to refuse. Concerns about adverse effects (p < 0.001) and beliefs that vaccination could encourage sexual activity (p = 0.01) also contributed to refusal, as did fears of vaccinating their daughters (p < 0.001), fear of regretting vaccination (p < 0.004), and fear of regretting non-vaccination (p < 0.004). External influences, including the negative impact of media (p = 0.001) and societal norms (p < 0.001), further affected parents’ decisions. Finally, children not vaccinated according to the schedule (p < 0.001), previous failure to follow vaccination schedules (p = 0.01), lack of information from health professionals about vaccine availability (p < 0.001) and free provision (p < 0.001), as well as non-recommendation of the HPV vaccine by health professionals (p < 0.001), were all significantly associated with parental refusal “Table 5”.

**Table 5:** Multivariate analysis (odds ratio, *P-value*) of risk factors for refusal of human papillomavirus vaccination among parents of young girls, Morocco, 2025.

	Bivariate analysis		Multivariate analysis	
	OR (95% CI)	*P-value*	AOR (95% CI)	*P-value*
Rural area of residence	1.49 [1.19-1.88]	0.0005	1.19 [0.78-1.82]	0.41
Being uncoupled	1.48 [1.00-2.18]	0.049	1.79 [0.99-3.23]	0.05
**Level of education**		0.001		
University/Illiteracy	1.11 [0.80-1.53]		3.88 [1.67-9.05]	0.001
High school/Illiteracy	1.58 [1.15-2.16]		3.24 [1.72-6.11]	0.0003
College/Illiteracy	1.82 [1.30-2.56]		3.45 [1.99-5.96]	<0.0001
Primary/Illiteracy	1.54 [0.91-2.60]		1.43 [0.85-2.40]	0.17
**Partner’s level of education**		0.0003		
University/ Illiteracy	0.74 [0.52-1.07]		1.72 [0.86-3.42]	0.12
High school/Illiteracy	1.45 [1.03-2.04]		1.84 [1.02-3.31]	0.04
Middle school/Illiteracy	1.66 [1.19-2.30]		1.07 [0.61-1.86]	0.80
Elementary/Illiteracy	1.29 [0.90-1.84]		1.03 [0.59-1.82]	0.89
**Monthly income per household ($)**		<0.001		
<199/ ≥ 500	2.27 [1.61-3.18]		2.26 [1.21-4.24]	0.01
200-299/ ≥ 500	1.03 [0.73-1.45]		1.38 [0.75-2.54]	0.29
300-499/ ≥ 500	0.85 [0.60-1.22]		1.11 [0.65-1.90]	0.69
**Time to reach health center**		0.0005		
> One hour/ < 30 min	3.64 [1.80-7.35]		6.49 [2.32-18.17]	0.0004
Between a 30 min and 1 hour / < 30 min	1.30 [0.96-1.76]		1.26 [0.74-2.12]	0.38
**Type of transport**		0.005		
Transport / on foot	0.72 [0.57-0.90]		0.71 [0.46-1.10]	0.13
**Trust in the healthcare system**		<0.001		
Lack of trust in the healthcare system/Trust in the healthcare system	28.3 [18.9-42.5]		28.4 [15.59-51.7]	<0.001
Low trust on healthcare system / Trust on healthcare system	6.12 [4.54-8.25]		3.82 [2.52-5.79]	<0.001
Not having previously heard of HPV	1.71 [1.32-2.22]	<0.001	1.37 [0.81-2.31]	0.23
Not having previously heard of cervical cancer	2.70 [2.13-3.42]	<0.001	2.67 [1.70-4.19]	<0.001
Having been in contact with a person who has cervical cancer	1.45 [1.06-1.98]	0.01	1.19 [0.71-1.99]	0.50
Insufficient knowledge of cervical cancer risk factors	2.68 [2.08-3.44]	<0.001	1.17 [0.74-1.83]	0.48
Insufficient knowledge of the symptoms of cervical cancer	4.41 [3.50-6.88]	<0.001	2.75 [1.58-4.78]	0.0003
Insufficient knowledge of the complication of cervical cancer	2.64 [1.77-3.94]	<0.001	0.85 [0.41-1.74]	0.66
Insufficient knowledge of the preventive measure of cervical cancer	2.96 [2.29-3.83]	<0.001	1.06 [0.64-1.78]	0.80
Insufficient knowledge about the number of doses of the HPV vaccine	0.61 [0.46-0.82]	0.0013	0.42 [0.25-0.70]	0.001
Low perception of the level of information provided by the media on the HPV vaccine	1.80 [1.33-2.42]	0.0001	2.03 [1.21-3.41]	0.007
Susceptibility to HPV infection No/Yes	0.35 [0.28-0.44]	<0.001	1.50 [0.92-2.45]	0.10
Low perception of HPV severity	4.50 [3.44-5.88]	<0.001	2.43 [1.37-4.30]	0.002
Low perception of HPV vaccine benefits	4.28 [3.34-5.49]	<0.001	1.34 [0.81-2.21]	0.24
HPV vaccine adverse reaction	0.13 [0.10-0.17]	<0.001	0.58 [0.37-0.89]	0.01
HPV vaccine would encourage sexual activity	0.73 [0.56-0.94]	0.01	1.07 [0.68-1.67]	0.76
Parents’ fear of having their young girls vaccinated with the anti-HPV vaccine	4.97 [3.85-6.42]	<0.001	3.98 [2.45-6.46]	<0.001
Parents’ fear of regretting having vaccinated their young girls with the anti-HPV vaccine	2.24 [1.78-2.83]	<0.001	1.25 [0.78-2.02]	0.33
Negative media influence on HPV vaccination	1.50 [1.17-1.93]	0.001	0.78 [0.43-1.39]	0.40
Negative influence of societal norms on HPV vaccination	2.33 [1.81-3.00]	<0.001	1.23 [0.72-2.09]	0.42
Not having already vaccinated your children previously according to the vaccination schedule	8.10 [5.20-12.6]	<0.001	3.86 [2.05-7.28]	<0.001
Having previously refused to have their children vaccinated according to the recommended vaccination schedule	6.26 [1.21-32.4]	0.01	0.39 [0.05-2.80]	0.35
The doctor or nurse does not inform the parents that the health center has a vaccine against HPV	2.38 [1.79-3.16]	<0.001	0.29 [0.12-0.70]	0.006
The doctor or nurse does not inform the parents about the free HPV vaccine at the health center	2.64 [1.99-3.50]	<0.001	0.86 [0.26-2.83]	0.80
The doctor or nurse doesn’t advise parents to vaccinate their daughters against HPV at the health center	3.11 [2.32-4.18]	<0.001	7.39 [2.24-24.3]	0.001

*HPV stands for human papillomavirus. AOR stands for Adjusted Odds Ratio, CI stands for confidence interval*

### 6. Multivariate analysis

After adjustment for potential confounders, 15 factors were independently associated with parental refusal of HPV vaccination. Refusal was associated with parents with a higher educational level, including university education (AOR = 3.88; 95% CI: 1.67–9.05), high school education (AOR = 3.24; 95% CI: 1.72–6.11), and college education overall (AOR = 3.45; 95% CI: 1.99–5.96), as well as among those whose spouse had a high school level of education (AOR = 1.84; 95% CI: 1.02–3.31). Socioeconomic and access-related factors were also significant, including low monthly household income (AOR = 2.26; 95% CI: 1.21–4.24) and a travel time exceeding one hour to reach a health center (AOR = 6.49; 95% CI: 2.32–18.17).

Healthcare system–related factors showed a strong association with refusal, particularly lack of trust in the healthcare system (AOR = 28.4; 95% CI: 15.59–51.7), low trust in the healthcare system (AOR = 3.82; 95% CI: 2.52–5.79), and absence of HPV vaccine recommendation by healthcare professionals (AOR = 7.39; 95% CI: 2.24–24.3).

In addition, knowledge- and perception-related factors were significantly associated with refusal, including never having heard of cervical cancer (AOR = 2.67; 95% CI: 1.70–4.19), insufficient knowledge of its symptoms (AOR = 2.75; 95% CI: 1.58–4.78), low perceived level of information provided by the media about the HPV vaccine (AOR = 2.03; 95% CI: 1.21–3.41), and low perceived severity of HPV infection (AOR = 2.43; 95% CI: 1.37–4.30). Behavioral factors such as fear related to vaccinating daughters against HPV (AOR = 3.98; 95% CI: 2.45–6.46) and non-adherence to previous childhood vaccination schedules (AOR = 3.86; 95% CI: 2.05–7.28) were also identified.

Conversely, several factors were found to be protective against vaccine refusal, including lack of knowledge regarding the number of required HPV vaccine doses (AOR = 0.42; 95% CI: 0.25–0.70), belief that the vaccine may cause adverse effects (AOR = 0.58; 95% CI: 0.37–0.89), and parents’ lack of awareness due to insufficient communication by healthcare professionals of HPV vaccine availability at health centers (AOR = 0.29; 95% CI: 0.12–0.70) “Table 5”.

## Discussion

Vaccination against HPV represents a cornerstone strategy for the prevention of cervical cancer. Nevertheless, persistent parental refusal since the introduction of the vaccine into national immunization programs has limited its uptake and reduced its potential benefits for women’s sexual and reproductive health. In our study, 28.7% of parents reported refusing HPV vaccination for their daughters. This finding is consistent with rates reported in the scientific literature, which range from 20% to 44%. For example, refusal rates of 43.2% were reported in China in 2024 among 11,728 participants [18]; 34.5% in Saudi Arabia in 2023 among 534 participants [19]; 27.9% in Pakistan in 2021 among 610 participants [20]; 22.1% in Ivory Coast in 2024 among 181 participants [21]; and 20.1% in India in 2020 among 831 participants [22].

A lack of trust in the healthcare system is a risk factor associated with vaccine refusal. The spread of alarmist or anti-vaccine messages, often fueled by social media, exacerbates this loss of trust and reinforces refusal [23]. Contexts marked by fragile relationships between healthcare professionals and patients worsen this dynamic [24].

The lack of HPV vaccine recommendations from healthcare professionals is a risk factor associated with vaccine refusal. This finding aligns with the results of the scientific literature [25,26]. Indeed, in the United States, parents who did not receive an explicit recommendation were more likely to refuse the HPV vaccine. Kata et al. showed that the lack of professional guidance leaves an information gap that is quickly filled by misinformation on social media, thus exacerbating refusal [23].

The time required to reach a health center is a risk factor for vaccine refusal. In our study, parents living more than an hour from the health center were significantly more likely to refuse the HPV vaccine for their daughters. This finding can be explained by logistical constraints and the cost of travel, which reduce accessibility and lower the priority of vaccination [27]. Epidemiological studies conducted in low- and middle-income countries report that geographical remoteness and a lack of reliable transportation are consistently associated with vaccine refusal [28,29].

In our study, fear of vaccinating girls against HPV was a risk factor for vaccine refusal. Parents may link this fear to their perception of health risks, including moral and cultural concerns surrounding vaccination against a sexually transmitted infection [25].

High parental education levels are a risk factor associated with HPV vaccine refusal. Indeed, some more educated parents are more frequently exposed to conflicting information or critical messages about the vaccine, which can reinforce mistrust and lead to refusal [30]. These parents often have heightened critical thinking skills and actively seek alternative information, which may lead them to question the necessity or safety of the HPV vaccine [31].

Failure to adhere to the childhood vaccination schedule is a risk factor for HPV vaccine refusal. Parents who have not vaccinated their children according to the recommended schedule appear more likely to refuse further vaccinations, probably due to general vaccine hesitancy, lack of awareness, or the influence of personal and cultural beliefs [30]. A study in the United States showed that parents who delayed or refused childhood vaccinations were significantly more likely to refuse the HPV vaccine for their adolescents [26]. In the MENA region, studies conducted in Tunisia and Saudi Arabia also report that omission or non-adherence to the vaccination schedule is associated with lower HPV vaccine acceptance [32].

In our survey, a lack of knowledge of cervical cancer symptoms was a risk factor associated with refusal of the HPV vaccine. That could be explained by the tendency of these parents to underestimate the severity of and vulnerability to HPV infection, which reduces the perceived need for vaccination [25].

Not having heard of cervical cancer is a factor associated with refusing the HPV vaccine. Indeed, parents who have never heard of this disease often believe their daughters are not at risk, leading to a refusal of vaccination [25].

Low household income is a significant factor associated with refusal of the HPV vaccine, even though it is offered free of charge. Indeed, even without direct financial costs, vaccination can involve hidden costs—organizational or time-related—that disproportionately affect low-income families and contribute to refusal. Thus, despite the vaccine being free, the social, cognitive, and institutional dimensions linked to low income are decisive factors in HPV vaccination refusal.

A low perception of the severity of HPV infection is a risk factor associated with vaccine refusal. Indeed, when parents underestimate the potential severity of HPV, particularly its causal link to cervical cancer, they are less inclined to consider vaccination a necessary measure. This relationship is well documented by the Health Belief Model, according to which a low perception of severity directly reduces the intention to adopt preventive behavior [33]. Several studies have confirmed that parents who consider HPV a benign infection have higher rates of vaccine refusal because they do not perceive the urgency or importance of immunizing their children [34]. Furthermore, a lack of awareness of the link between HPV and cancer contributes to the trivialization of the infection, a phenomenon described in many contexts, particularly in the Middle East and North Africa [35].

An unsatisfactory perception of information provided by the media about the HPV vaccine is a risk factor associated with vaccine refusal. Indeed, when media messages are deemed insufficient, unclear, or lacking in credibility, parents more readily develop doubts about the vaccine’s efficacy and safety, thus reinforcing their refusal. Several studies show that the quality and clarity of media information play a central role in vaccine acceptance. A lack of clear communication increases mistrust and promotes exposure to unreliable sources disseminating anxiety-inducing or erroneous messages [36]. Research conducted in various settings has also demonstrated that parents who feel the media do not provide sufficient or consistent information are significantly more likely to refuse the HPV vaccine [37,38]. Because our measure aggregated all media sources, we cannot determine whether negative influence was driven primarily by social media misinformation or by traditional media underreporting. Future research should disaggregate these channels.

The lack of clear recommendations for the HPV vaccine from healthcare professionals constitutes a significant risk factor for HPV vaccine refusal. This may be explained by the central role of healthcare professionals as parents’ most trusted source of information regarding vaccination. Evidence from Gilkey et *al.* demonstrates that strong, clear, and timely recommendations markedly increase HPV vaccine acceptance, whereas weak or absent recommendations foster uncertainty, fear, and ultimately refusal [39].

The scientific literature shows that perceiving vaccine side effects as mild, expected, and non-dangerous promotes vaccine acceptance. Transparency about mild post-vaccination reactions strengthens parental confidence and reduces uncertainty [40]. Parents who perceive vaccine side effects as typical are less inclined to decline vaccination, indicating a pragmatic comprehension of vaccine safety. However, the finding that fear of adverse effects was more frequently reported among acceptors remains paradoxical and cannot be fully explained by knowledge alone. This may reflect a social desirability bias, whereby participants who accepted vaccination felt inclined to acknowledge potential risks without allowing these concerns to influence their decision. In contrast, those who refused vaccination may have minimized or dismissed these risks as a way to justify their choice.

Unexpectedly, a lack of knowledge regarding the recommended number of HPV vaccine doses emerged as a protective factor in our analysis. This result may seem counterintuitive, given that the literature generally associates low levels of knowledge with lower vaccine acceptance [41]. One possible explanation lies in the fact that vaccine acceptance depends not only on detailed factual knowledge but also on factors such as trust in healthcare professionals and general attitudes toward vaccination. Studies have indicated that a healthcare professional’s recommendation is one of the most influential determinants of adherence to HPV vaccination, regardless of specific knowledge level [39]. Thus, participants who were unfamiliar with the precise vaccination schedule might still adopt a favorable attitude by relying on medical recommendations.

Similarly, a lack of awareness regarding the availability of the HPV vaccine at health centers emerged as a protective factor against vaccine refusal. This seemingly paradoxical result contrasts with data from the literature suggesting that access to information and the perception of service availability generally promote vaccination adherence [25]. Several hypotheses can be put forward to explain this association. Indeed, vaccine acceptance often relies more on relational and contextual factors, particularly trust in healthcare professionals and the strength of their recommendation, than on logistical knowledge of services. It has been widely demonstrated that a healthcare professional’s recommendation is one of the most influential factors in the decision to receive HPV vaccination [39], which could explain why participants unaware of vaccine availability nevertheless remain willing to receive it when offered. Similarly, partial awareness of vaccine availability could lead to misinterpretations or concerns about actual accessibility (cost, continuity of supply, quality of services), potentially increasing vaccine hesitancy. Thus, it is not so much the information itself, but how it is understood and integrated, that could influence attitudes.

## Limitations

Our study has various limitations, the most prominent of which is the cross-sectional design. While this method is often appreciated for its speed, simplicity, and cost-effectiveness, it does not allow us to establish a strong causal relationship between exposure variables and rejection of HPV vaccination because all data are collected at a single time point. Furthermore, household income data should be interpreted with caution, as they may have been influenced by prevarication bias, with some participants potentially underreporting or misreporting their income. Sexual activity status was based solely on parental self-reporting and may be subject to misclassification bias due to the sensitive nature of the topic. The influence of media on parental perception was assessed globally, encompassing both traditional media and social media, but the specific impact of each type of media was not distinguished.

## Conclusion

Vaccination against HPV remains an essential strategy for preventing cervical cancer. However, persistent vaccine refusal limits its impact. In our study, numerous risk factors were identified, including low trust in the healthcare system, fear related to vaccination, lack of knowledge of cervical cancer symptoms, reduced perception of the infection’s severity, failure to adhere to the previous vaccination schedule, low household income, and the absence of explicit recommendations from healthcare professionals. These findings highlight the need for targeted public health interventions to raise awareness about HPV and its vaccination, with particular emphasis on empowering healthcare professionals to actively provide clear guidance to parents. Communication strategies should be developed in a targeted and practical manner, including mandatory training for healthcare professionals and information sessions at health centers, community gathering places (e.g., women’s associations, mosques), and schools, tailored to the recommended vaccination ages and parents’ educational levels. Conversely, some protective factors, such as the perception of mild side effects, lack of knowledge about the exact number of doses required, and a lack of information on vaccine availability at the health center, were associated with vaccine acceptance by enabling parents to make a more autonomous and pragmatic decision. These results highlight the need for comprehensive strategies that include clear communication, culturally sensitive awareness campaigns, and active involvement of healthcare professionals. Promoting trust, addressing misconceptions, and supporting families in their decisions are essential to increasing HPV vaccine acceptance and safeguarding the sexual and reproductive health of women.

## Supporting information

S1 File Inclusivity in global research questionnaire. (DOCX)

## References

[1] Zach R, Bentwich ME. Reasons for and insights about HPV vaccination refusal among ultra-Orthodox Jewish mothers. Dev World Bioeth. 2023;23(4):300–11. doi: 10.1111/dewb.12372 36201654

[2] IARC marks cervical cancer awareness month 2025. IARC. Accessed 2025 November 30. https://www.iarc.who.int/news-events/iarc-marks-cervical-cancer-awareness-month-2025/

[3] Cancer du col de l’utérus. Accessed 2025 December 16. https://www.who.int/fr/news-room/fact-sheets/detail/cervical-cancer

[4] World Health Organization Regional Office for Africs. Cervical cancer. World Health Organization. Accessed 2025 November 30. https://www.afro.who.int/health-topics/cervical-cancer

[5] Stratégie régionale d’élimination du cancer du col de l’utérus dans la Méditerranée orientale. 2023.

[6] Complete cervical cancer profile sets. Accessed 2025 December 16. https://www.who.int/publications/m/item/cervical-cancer-country-profiles

[7] Initiative pour elimination du cancer du col de uterus. Accessed 2025 December 17. https://www.who.int/fr/initiatives/cervical-cancer-elimination-initiative

[8] Plan national du cancer : prévention et contrôle. 2020.

[9] Zraidi M, Ibriz M. Human papillomavirus vaccination in view of the National Cancer Control Plan 2020-2029 in Morocco. Pan Afr Med J. 2023;45:115. doi: 10.11604/pamj.2023.45.115.40004 37745922 PMC10516758

[10] Aldossary MS, Mufrrih M, El Dalatony MM, Alamri HM. Prevalence and genotypes’ distribution of human papillomavirus among women in Saudi Arabia: a systematic review and meta-analysis. Front Public Health. 2025;13:1580699. doi: 10.3389/fpubh.2025.1580699 40443939 PMC12119489

[11] Rezq KA, Algamdi M, Alanazi R, Alanazi S, Alhujairy F, Albalawi R, et al. Knowledge, perception, and acceptance of hpv vaccination and screening for cervical cancer among Saudi Females: a cross-sectional study. Vaccines (Basel). 2023;11(7):1188. doi: 10.3390/vaccines11071188 37515004 PMC10385735

[12] Ahmed HAA, Abbas MH, Hussein HA, Nasr RSF, Lashen AA, Khaled H, et al. Cervical cancer screening uptake in Arab countries: a systematic review with meta-analysis. BMC Cancer. 2024;24(1):1438. doi: 10.1186/s12885-024-13204-7 39574088 PMC11583763

[13] Offre de soins de santé. Accessed 2025 March 20. http://cartesanitaire.sante.gov.ma/dashboard/pages2/index_2024.html

[14] Essayagh T, Essayagh M, Essayagh S. Drug non-adherence in hypertensive patients in Morocco, and its associated risk factors. Eur J Cardiovasc Nurs. 2021;20(4):324–30. doi: 10.1093/eurjcn/zvaa002 33620474

[15] Belayachi S, Boukhari FZ, Essayagh F, Terkiba O, Zohoun A, Essayagh M, et al. Non-adherence to antihypertensive drugs and its risk factors among hypertensive patients, Marrakech, Morocco. PLOS Glob Public Health. 2024;4(8):e0002774. doi: 10.1371/journal.pgph.0002774 39190663 PMC11349104

[16] Belayachi S, Boukhari FZ, Essayagh F, Terkiba O, Marc I, Lambaki A, et al. Uncontrolled blood pressure and its risk factors among hypertensive patients, Marrakech, Morocco. Sci Rep. 2024;14(1):2953. doi: 10.1038/s41598-024-53115-y 38316867 PMC10844197

[17] Essayagh T, Essayagh M, El Rhaffouli A, Khouchoua M, Bukassa Kazadi G, Khattabi A, et al. Prevalence of uncontrolled blood pressure in Meknes, Morocco, and its associated risk factors in 2017. PLoS One. 2019;14(8):e0220710. doi: 10.1371/journal.pone.0220710 31398197 PMC6688818

[18] Lin Z, Chen S, Su L, Chen H, Fang Y, Liang X, et al. Exploring mother-daughter communication and social media influence on HPV vaccine refusal for daughters aged 9-17 years in a cross-sectional survey of 11,728 mothers in China. Hum Vaccin Immunother. 2024;20(1):2333111. doi: 10.1080/21645515.2024.2333111 38530324 PMC10968318

[19] Turki YM, Alqurashi J. Knowledge, attitudes, and perceptions towards human papillomavirus (HPV) vaccination among adult women in primary health care centers in Makkah, Saudi Arabia. Cureus. 2023;15. doi: 10.7759/CUREUS.44157 PMC10460136 37638260

[20] Khattak FA, Rehman K, Shahzad M, Arif N, Ullah N, Kibria Z, et al. Prevalence of parental refusal rate and its associated factors in routine immunization by using WHO vaccine hesitancy tool: a cross sectional study at district Bannu, KP, Pakistan. Int J Infect Dis. 2021;104:117–24. doi: 10.1016/j.ijid.2020.12.029 33340667

[21] Kounangui MNAA, Kouadio DE, Assamala ME, Loukou GK, Bangaman CA, Kouassi C. Déterminants du refus de la vaccination contre le papillomavirus humain chez les parents des enfants de 9 à 14 ans au sein des ménages d’Aliodan en 2024, Côte d’Ivoire: étude transversale quantitative. PAMJ. 2025;51:62. doi: 10.11604/PAMJ.2025.51.62.46844 41064670 PMC12500871

[22] Degarege A, Krupp K, Fennie K, Srinivas V, Li T, Stephens DP, et al. Human papillomavirus vaccine acceptability among parents of adolescent girls in a rural area, Mysore, India. J Pediatr Adolesc Gynecol. 2018;31(6):583–91. doi: 10.1016/j.jpag.2018.07.008 30055285 PMC7679173

[23] Kata A. Anti-vaccine activists, Web 2.0, and the postmodern paradigm--an overview of tactics and tropes used online by the anti-vaccination movement. Vaccine. 2012;30(25):3778–89. doi: 10.1016/j.vaccine.2011.11.112 22172504

[24] Yaqub O, Castle-Clarke S, Sevdalis N, Chataway J. Attitudes to vaccination: a critical review. Soc Sci Med. 2014;112:1–11. doi: 10.1016/j.socscimed.2014.04.018 24788111

[25] Holman DM, Benard V, Roland KB, Watson M, Liddon N, Stokley S. Barriers to human papillomavirus vaccination among US adolescents: a systematic review of the literature. JAMA Pediatr. 2014;168(1):76–82. doi: 10.1001/jamapediatrics.2013.2752 24276343 PMC4538997

[26] Gilkey MB, McRee A-L, Magnus BE, Reiter PL, Dempsey AF, Brewer NT. Vaccination confidence and parental refusal/delay of early childhood vaccines. PLoS One. 2016;11(7):e0159087. doi: 10.1371/journal.pone.0159087 27391098 PMC4938536

[27] LaMontagne DS, Barge S, Le NT, Mugisha E, Penny ME, Gandhi S, et al. Human papillomavirus vaccine delivery strategies that achieved high coverage in low- and middle-income countries. Bull World Health Organ. 2011;89(11):821-830B. doi: 10.2471/BLT.11.089862 22084528 PMC3209730

[28] Cleland J, Bernstein S, Ezeh A, Faundes A, Glasier A, Innis J. Family planning: the unfinished agenda. Lancet. 2006;368(9549):1810–27. doi: 10.1016/S0140-6736(06)69480-4 17113431

[29] Oyo-Ita A, Oduwole O, Arikpo D, Effa EE, Esu EB, Balakrishna Y, et al. Interventions for improving coverage of childhood immunisation in low- and middle-income countries. Cochrane Database Syst Rev. 2023;12(12):CD008145. doi: 10.1002/14651858.CD008145.pub4 38054505 PMC10698843

[30] Gust DA, Darling N, Kennedy A, Schwartz B. Parents with doubts about vaccines: which vaccines and reasons why. Pediatrics. 2008;122(4):718–25. doi: 10.1542/peds.2007-0538 18829793

[31] Brewer NT, Fazekas KI. Predictors of HPV vaccine acceptability: a theory-informed, systematic review. Prev Med. 2007;45(2–3):107–14. doi: 10.1016/j.ypmed.2007.05.013 17628649

[32] Fallatah DI, Khalil MA, Abd ElHafeez S, Gouda S, Alshanbari HM, Awadalla M, et al. Factors influencing human papillomavirus vaccine uptake among parents and teachers of schoolgirls in Saudi Arabia: a cross-sectional study. Front Public Health. 2024;12:1403634. doi: 10.3389/fpubh.2024.1403634 39494075 PMC11528711

[33] Rosenstock IM, Strecher VJ, Becker MH. Social learning theory and the Health Belief Model. Health Educ Q. 1988;15(2):175–83. doi: 10.1177/109019818801500203 3378902

[34] Brewer NT, Fazekas KI. Predictors of HPV vaccine acceptability: a theory-informed, systematic review. Prev Med. 2007;45(2–3):107–14. doi: 10.1016/j.ypmed.2007.05.013 17628649

[35] Almazrou S, Saddik B, Jradi H. Knowledge, attitudes, and practices of Saudi physicians regarding cervical cancer and the human papilloma virus vaccine. J Infect Public Health. 2020;13(4):584–90. doi: 10.1016/j.jiph.2019.09.002 31570271

[36] Hopfer S. Effects of a narrative HPV vaccination intervention aimed at reaching college women: a randomized controlled trial. Prev Sci. 2012;13(2):173–82. doi: 10.1007/s11121-011-0254-1 21993613

[37] Viswanath K, Bekalu M, Dhawan D, Pinnamaneni R, Lang J, McLoud R. Individual and social determinants of COVID-19 vaccine uptake. BMC Public Health. 2021;21(1):818. doi: 10.1186/s12889-021-10862-1 33910558 PMC8081000

[38] Cates JR, Shafer A, Carpentier FD, Reiter PL, Brewer NT, McRee A-L, et al. How parents hear about human papillomavirus vaccine: implications for uptake. J Adolesc Health. 2010;47(3):305–8. doi: 10.1016/j.jadohealth.2010.04.003 20708571 PMC2928162

[39] Gilkey MB, McRee A-L. Provider communication about HPV vaccination: a systematic review. Hum Vaccin Immunother. 2016;12(6):1454–68. doi: 10.1080/21645515.2015.1129090 26838681 PMC4964733

[40] Larson HJ, Jarrett C, Eckersberger E, Smith DMD, Paterson P. Understanding vaccine hesitancy around vaccines and vaccination from a global perspective: a systematic review of published literature, 2007-2012. Vaccine. 2014;32:2150–9. doi: 10.1016/J.VACCINE.2014.01.081 24598724

[41] Kessels SJM, Marshall HS, Watson M, Braunack-Mayer AJ, Reuzel R, Tooher RL. Factors associated with HPV vaccine uptake in teenage girls: a systematic review. Vaccine. 2012;30(24):3546–56. doi: 10.1016/j.vaccine.2012.03.063 22480928

